# Estimation of the Age and Reproductive Performance of Wild-Born and Escaped Mink (*Neovison vison*) Caught in the Wild in Denmark

**DOI:** 10.3390/ani11010162

**Published:** 2021-01-12

**Authors:** Sussie Pagh, Cino Pertoldi, Mariann Chriel, Heidi Huus Petersen, Trine Hammer Jensen, Sussi Madsen, David Chr. Evar Kraft, Toke Munk Schou, Mette Sif Hansen

**Affiliations:** 1 Department of Chemistry and Bioscience—Section of Biology and Environmental Science, Aalborg University, Fredrik Bajers Vej 7, 9220 Aalborg, Denmark; cp@bio.aau.dk (C.P.); thj@bio.aau.dk (T.H.J.); tokeschou@gmail.com (T.M.S.); 2 Aalborg Zoo, Mølleparkvej 63, 9000 Aalborg, Denmark; 3 National Veterinary Institute, Technical University of Denmark, Kemitorvet, 2800 Kgs. Lyngby, Denmark; machr@mst.dk (M.C.); hhpet@dtu.dk (H.H.P.); 4 Department of Dentistry and Oral Health, University of Aarhus, Vennelyst Boulevard 9, 8000 Aarhus C, Denmark; sussi.madsen@dent.au.dk (S.M.); dck@dent.au.dk (D.C.E.K.); 5 Sektion of Pathobiology, University of Copenhagen, Ridebanevej 3, 1870 Frederiksberg C, Denmark; msha@sund.ku.dk

**Keywords:** placental scars, demography, litter size, fecundity, turnover

## Abstract

**Simple Summary:**

Previous studies of wild caught mink in Denmark showed that 30–80% had recently escaped from farms. Therefore, it is debated whether a self-sustaining feral mink population is established in Denmark or whether the population rests upon a continuous contribution of captive-born farm mink. Knowledge regarding the reproduction and mortality of mink adapted for living in the wild is important for the management of feral mink. In this study, we separated wild-born from captive-born mink caught in the wild in Denmark. To be able to age the mink in this study, an age determination model for mink was developed based on the width of the pulp cavity. The mean litter size of wild-born female mink was 7.6 (range: 5–11 kits). The annual turnover of mink caught in the wild was estimated to be 66%, and the yearly mortality was 69%; thus, the population of wild-born mink is slightly declining. The results confirmed that the wild-born mink population in Denmark is reproducing and self-sustaining without a continuous influx of captive-born mink escaped from farms. The effect of escaped mink on the wild mink population will depend on the currently unknown ability of captive mink to survive in the wild.

**Abstract:**

The feral mink population in Denmark consists of two groups of animals: mink born in the wild and mink that have recently escaped from farms. The aims of this study were to: (1) estimate the reproductive performance and mortality of the Danish mink born in the wild (wild-born) and mink escaped from farms (captive-born); (2) discuss the likelihood of a self-sustaining population of wild-born mink in Denmark; and (3) model the relationship between the pulp cavity width and the age of mink. During 2018, 247 wild caught mink were sent for necropsy at the Danish National Veterinary Institute. Based on body length, 112 were determined as captive-born and 96 as wild-born. The mean litter size ± SE of wild-born females was 7.6 ± 0.9 (range: 5–11 kits) and for captive-born females 5.9 ± 0.9 (range: 1–10 kits). The relationship between age (in months) of mink and pulp cavity width was highly significant. Individuals with a pulp cavity width of >35% were younger than one year. Based on fecundity, the turnover of the mink population was estimated to be 66%, and the yearly mortality was estimated at 69%. Hence, the population is slightly declining. In conclusion, a feral reproducing mink population in Denmark persists without a continuous influx of captive-born mink from farms.

## 1. Introduction

**Figure 1:**
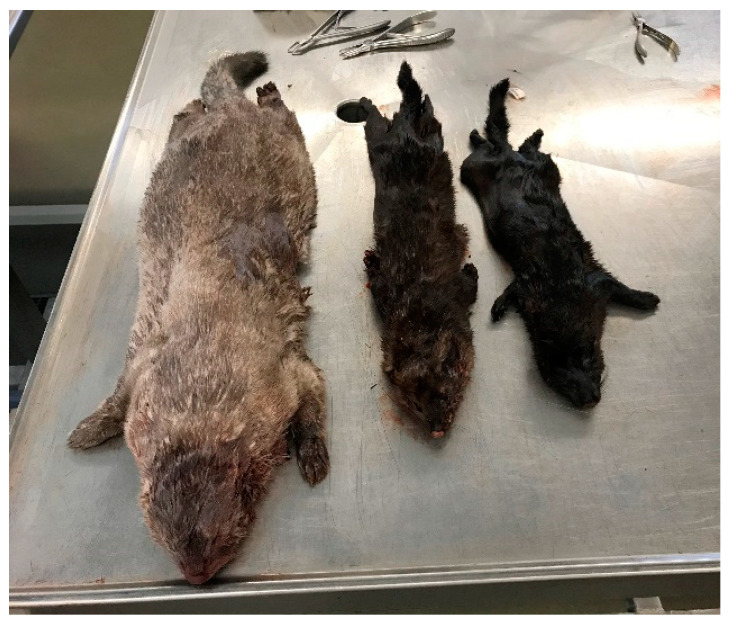
Male captive-born (left), male wild-born (middle), and female wild-born (right) mink.

Both accidental and intentional introductions of alien species into nature may have a large impact on native ecosystems [1]. The American mink (*Neovison vison*) is a semi-aquatic medium sized carnivore, native to North America [2]. The population of American mink in Denmark remained relatively small until the late 1980s, where after the annual hunting of mink increased from around 1000 to 8000 mink near the millennium. In 2018, the national game bags of mink were less than 2000 mink [3]. Mink have been bred for their fur in Denmark since the mid-1920s [4]. During the years of study, there were around 1300 commercial mink farms in Denmark producing around 17 million pelts per year [5]. Mink are considered as an invasive species in Denmark and are culled by hunters throughout the year [6]. They can be found in most parts of Denmark due to escapes from Danish farms [6]. The size of farmed mink has increased markedly over time, and they can therefore be separated from the much smaller wild-born mink. In Denmark, both breeding males and females have increased their mean weight by 70% for the past 10–15 years [7] (Figure 1).

In previous Danish studies, 80% of the mink in the years 1998–2000 and approximately 30% mink caught in nature during the winters of 2014–2018 were found to be mink recently escaped from farms, leading to the question of whether a truly feral mink population exists in Denmark [7,8,9,10]. Feral mink in Denmark are regarded as a pest and are trapped throughout the year by dedicated hunters.

Studies of reproduction on wild-born mink inside and outside their native domain are scarce. No studies from Scandinavia previously documented the litter size of wild-born mink. Previous efforts to determine the litter size from placental scars in mink showed that the scars disappear a few months post-partum [11,12,13]. However, when using an iron staining technique on the uterine tissue, the implantation site is visible up to 7 months after parturition [13]. Litter size in wild mink from France, Spain, and Belarus [13,14] ranged between four and seven kits. In Scotland, the mean litter size of feral mink was 5.5 kits, and the percent-reproducing females of the adult population was 81% [15].

In Denmark, mink kits on farms are born between April 20th and May 15th with an average weaned litter size of 5.5 kits and a maximum litter size at birth of 17 kits [5,16,17]. Mink kits born in nature in Denmark are likewise born in April and May [18]. In the southern latitudes (Belarus), most females caught by the end of March were with embryos, and new-born mink kits were found in late April to the beginning of June [19].

The ages of individual carnivores, including mink, are generally determined using the widths of the pulp cavity and/or incremental lines in the canine teeth [20,21,22]. Growth of the pulp cavity and incremental lines in the canine teeth of carnivores is species-specific, and incremental lines in mink are challenging to interpret. For example, in a study of wild-born mink from Hokkaido, no incremental lines were observed in several individuals, assessed as adults [23]. Recent papers on age determination of mink refer to either Helldin (1997) [21] or the Matson’s Laboratory (P.O. Box 308, Milltown, MT 59851, USA) [15,24,25,26]. However, the model by Helldin (1997) was based on the width of the pulp cavity in the teeth of Eurasian pine martens (*Martes martes*). A model describing the relationship between the pulp cavity growth rate and the age of the individual has not previously been presented for mink.

To the best of our knowledge, there have been no attempts to separate wild living and newly escaped mink in other studies [5,16,17]. Due to the high number of captive-born mink caught in the wild in Denmark, it is crucial to separate captive-born from wild-born mink to study the reproduction and demography of the feral Danish mink. Knowledge regarding the fecundity and mortality of the wild living mink is crucial to understanding how well adapted American mink are to living in the wild in Denmark.

The aims of this study were to: (1) estimate the reproductive performance and mortality of the Danish mink born in the wild (wild-born) compared to mink escaped from farms (captive-born); (2) discuss the likelihood of a self-sustaining population of wild-born mink in Denmark; and (3) estimate the relationship between the pulp cavity width and the age of mink, as a species-specific tool for estimating the age of mink caught in the wild.

## 2. Materials and Methods

Mink culled by hunters from different regions of Denmark were sent for necropsy to the National Veterinary Institute of Denmark as part of a permanent Wildlife Disease Surveillance Program. Mink for this study were sampled from the 1st of March to November 2018. Few mink in the sample were culled and stored by hunters before this period. Most hunters use traps where mink are put down within 12 h after the mink has been trapped (according to legislation). The carcasses were kept at −20 °C until necropsy. At necropsy, the sex was recorded, the body length from nose tip to first vertebrae of the tail was measured, and the uteri horns with ovaries were removed as well as two canine teeth for age determination. All samples were kept at −20 °C until examination. Fur colors were determined by the use of color templates with color types known from farms [16]. Hunters were asked to provide the dates of mortality of individual mink.

### 2.1. Method for Separating Wild Mink from Captive Mink

In a previous study of Danish mink, both univariate analyses and Gaussian mixture model analysis demonstrated clear divisions between the body length of farmed mink and wild-born mink [7]. Mixture analysis identified two groups of the wild caught mink, one assigned to farmed mink (born in captivity) and another group of smaller mink born in the wild [7]. Hence, the mink sampled for the present study were separated into two groups: mink born in the wild (wild-born) and mink born on farms (captive-born) according to [7].

Wild-born and captive-born mink can be separated using body length when they are more than 4 months old. Considering that births occur between April and May, we were able to distinguish mink captured after the 1st of September between the two groups [7]. In this study, the mink were evaluated for age, and males and females more than 4 months old with body lengths of over 43 and 39 cm, respectively, were grouped as captive-born.

### 2.2. Method for the Age Determination of Mink

The age was estimated using the width of the pulp cavity and dental lines of the canine teeth bearing in mind that mink are born in April and May [21,22]. As dentine is deposited inward into the pulp cavity, gradually filling the cavity (centripetal growth), a wide pulp cavity is only observed in young animals [21]. Cementum, which forms the dental lines, is deposited over the root dentine (centrifugal growth). In mink, the first age line is normally observed when mink are 6–10 months old, i.e., from October to December. Thereafter, a line is produced each year. Hence, it is feasible to determine age with an interval of 1 year per age category [20,22,23].

To determine the mink age, the canine teeth were extracted, fixed for 36 h in 4% *w*/*v* formaldehyde solution, and dried overnight at 40 °C. Then, the teeth were embedded in cold-polymerizing metylmethacrylate-based resin. Saw sections of 70–100 µm were prepared 2 and 3 mm from the apex of the tooth root with a Leiden saw (Meprotech, Holland Heerhugowaard, The Netherlands). The unstained saw sections were examined with a fully automated Olympus BX61 microscope (Olympus Ltd., Ballerup, Denmark) equipped with a DP80 camera (Olympus Ltd., Ballerup, Denmark).

To correlate the pulp width in mink teeth versus age, the width of the canine tooth and width of pulp cavity was measured at the widest place (2–3 mm from the apex) of the canine, using Olympus cellSens Dimension software measuring to the nearest 1/1000 mm. Pulp width as a percentage of tooth width was plotted against mink age estimated from birth date set to April 1st. Two farmed females, known to be 2 years old, were used to estimate the width of the pulp cavity in mink of more than one year of age.

A model for the pulp cavity width versus age (months) was estimated through two steps: (1) The overall best fitting regression line was made, and (2) a new, best fitting regression line was made removing mink considered to be from the previous season (seen as outliers).

**Figure 2:**
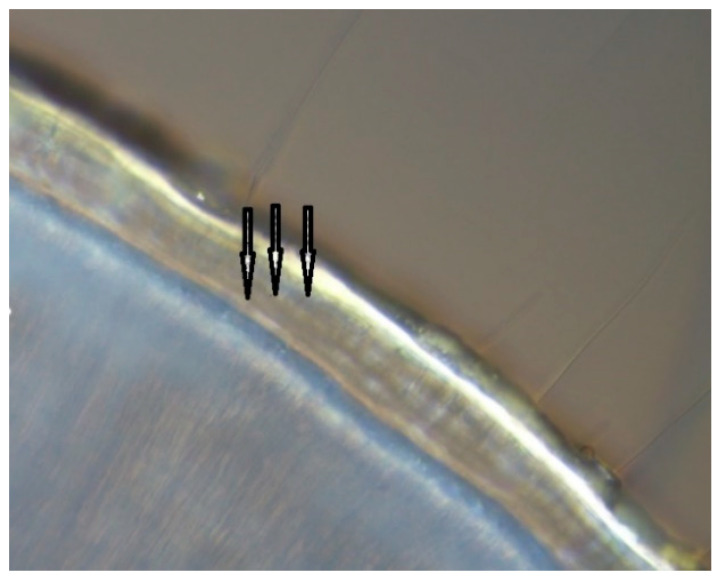
Saw sections (70–100 µm) of canine teeth of mink 2 and 3 mm from the apex under a microscope in reflected light differential interference contrast (DIC) microscopy. Three dark incremental lines (arrows) are visible.

The incremental lines were counted in individuals more than one year of age (based on percent width of the pulp cavity). After testing different microscope settings, reflected light differential interference contrast (DIC) microscopy was found to be optimal for obtaining high-magnification images of mink incremental lines (DIC cube U-MDIC3 and high-contrast DIC prism U-DICTHC, Olympus Ltd., Ballerup, Denmark; Figure 2).

### 2.3. Determination of the Reproductive Performance in Mink

**Figure 3:**
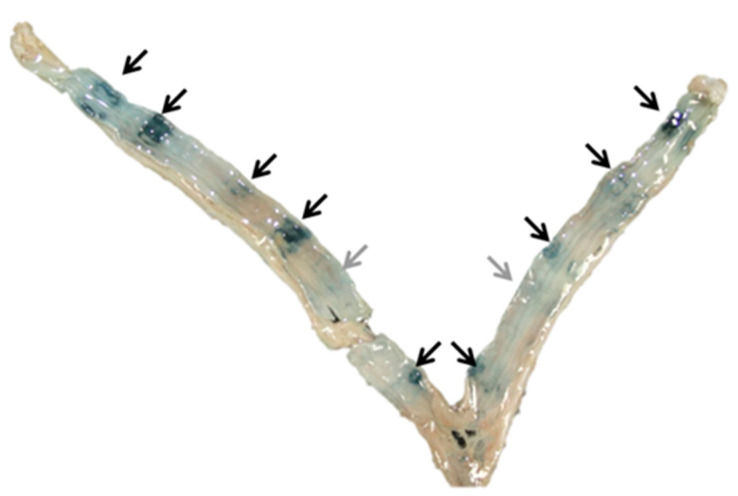
Uterus from a female mink with different staining intensity of placental scars (PSCs). Nine clearly visible PSCs (black arrows) and two pale scars (grey arrows).

The litter size of reproducing females, fecundity (the mean litter size of all adult females in the population, including barren), and proportion of reproducing/barren females were used to estimate the reproductive performance. The litter size was estimated from counts of the placental scars (PSCs) and embryos in the uterine horns (Figure 3).

In this study, the staining method described by [13] was used. The method identifies iron (from hemoglobin at the placental implantation site) in the uterine wall by dark blue staining (Figure 3). The uteri were thawed in tap water, followed by lengthwise opening of the uterine corpus and horns with scissors. The uteri were immersed in a fresh 10% solution of ammonium sulfide (H_8_N_2_S) for 10 min and rinsed in tap water, followed by a 10 min immersion in a 1:1 solution of 1% chlorhydric acid and 20% potassium hexacyanoferrate (K_4_(Fe(CN)_6_), 3H_2_O), rinsed in tap water and examined under a stereo microscope.

Females aged 0–10 months were classified as juveniles. Females 11–22 months old were classified as age class 1 (first season breeding females), whereas older females were classified as age-class 2, 3, and 4+. One-year-old or older females without reproductive activity, i.e., no PSCs or embryos, were characterized as barren.

Litter size is given for the reproducing females only, whereas the measure for fecundity expressed as the mean litter size included barren females. The fecundity and percent reproducing females was based only on females known to have been caught within 7 months after parturition (set to April 1st) to prevent an underestimation of fecundity and percent breeding females due to disappearance of the PSC after 7 months post-partum [13]. Throughout, the kits’ day of birth was set to April 1st, and females were expected to conceive at 10 months old [15]. The fecundity and barrenness was based on adult females euthanized between the 1st of March and 1st of November.

### 2.4. Statistical Analysis

T-tests were used to compare the reproduction of wild-born and captive-born-mink. Linear regression (least square) and polynomial regression was used to test the relationship between the pulp cavity width and age of the mink. Mean ± standard error (SE) is given for the litter size and fecundity.

The mortality rate = q(x) is the proportion of the population dying, i.e., the difference between the number of individuals found in different age groups in the collected population of mink. Turnover of the population, i.e., the percent of individuals that the population can tolerate to lose to mortality before the population will decrease, is based on the production (fecundity) of the population following Lloyd et al. (1976) [27].

The software program PAST was used for all statistical analyses: https://folk.uio.no/ohammer/past/.

### 2.5. Ethics Approval

All applicable international, national, and institutional guidelines for the care and use of animals were followed. No ethical approval was required from an institutional or national ethics review board. The study complied with current Danish laws. The research was carried out as part of the regular culling program for mink and the surveillance of wildlife diseases by the National Veterinary Institute Danish Technical University, Section for Diagnostics and Scientific Advice, Copenhagen.

## 3. Results

During 2018, 247 mink were submitted to the National Veterinary Institute of Denmark. Of these mink, 208 were separated as belonging to either the group of wild-born or captive-born; 96 mink were considered to have been born in the wild, and 112 mink were considered to have been born in captivity. Six mink out of 84 (7%) with known color in the wild-born group were recessive colored, while 43 out of 93 (46%) of the captive-born group with known color were recessive colored.

### 3.1. Age Determination

**Figure 4:**
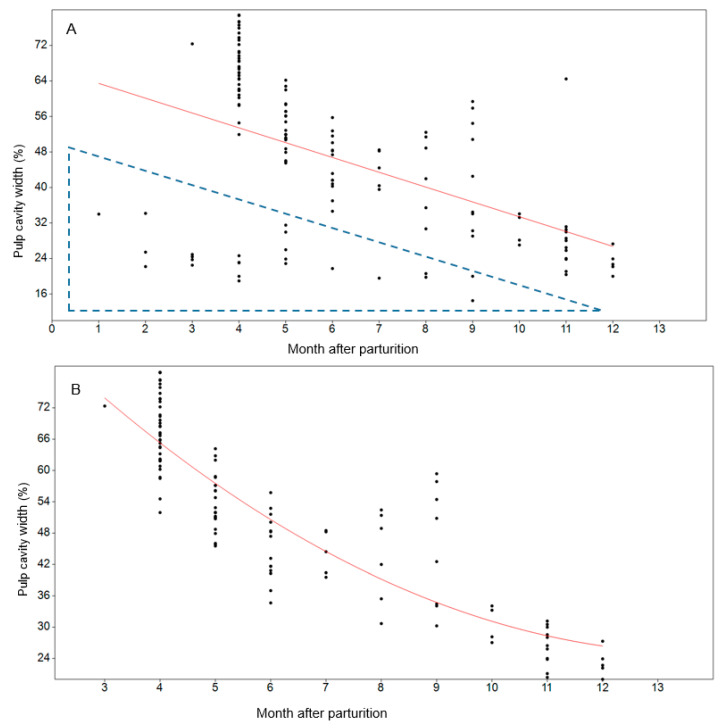
Pulp cavity width in relation to months after parturition. (**A**) All mink with a known death date (*n* = 141). The dotted triangle marks outliers, i.e., individuals with a pulp width of less than 35% before 6 months and individuals with a pulp width less than 25% considered to be born in the years before. (**B**) Polynomial regression line modeling the pulp cavity width of individuals < one year old in relation to month after parturition was y = 0.42x^2^ − 11.52x + 104.7, R^2^ = 0.77, *p* < 0.0001.

The exact mortality date was provided for 141 mink. For these individuals, the pulp cavity width ranged between 15% and 79% (Figure 4A). Pulp cavity is generally known to fill up with dentine along with the age of the individual. Individuals from the previous season will therefore confuse results within the first year. The pulp width of two farmed females, known to be two years of age, were 15% and 19%. Thus, individuals with a pulp cavity <20% were considered to be in their second year. Based on this, all individuals with a pulp cavity width of <25% were considered to be mink more than 12 months old, i.e., from the previous season. Mink that had died before 6 months post-parturition with a pulp width of less than 35% were considered to be from the previous season (see dotted triangle in Figure 4A). The best fitting regression line for mink less than 12 months old was y = 0.42x^2^ − 11.52x + 104.7, R^2^ = 0.77, *p* < 0.0001 (Figure 4B). The model-estimated pulp cavity at 10 months was 30%, ranging between 27% and 34%. Hence, individuals with a pulp cavity width of >35% were considered to be within their first year and not reproducing.

### 3.2. Reproduction

The uteri from 105 females were analyzed. Based on the pulp cavity width, 41 were adult females (pulp cavity <35%), and 64 were young females (<10 months, pulp width ≥ 34%). Young females <10 months were removed from the fecundity calculations. Of the 41 reproductively mature females, 34 females were culled between March 1st and November 1st. Hence, the lack of PSCs among females caught between March and November is not expected to be due to degeneration of scars, as with females caught after November. The mean litter size of females determined as wild-born (*n* = 7) was 7.6 ± 0.9, and the litter size of females considered to be captive-born (*n* = 11) was 5.9 ± 0.9 (Table 1). The fecundity of wild-born and captive-born females was 3.8 ± 1.2 and 3.4 ± 0.9, respectively (Table 1). The percent reproducing wild-born females was 50%, and that of captive-born was 58% (Table 1). No significant difference in the litter size (*t* = 1.25, *p* > 0.05) or fecundity (*t* = 0.03, *p* > 0.05) was found between wild-born and captive-born females; however, this may be due to small sample size and a large variance in litter size of captive-born females (Table 1).

No females with pulp width >35% showed any signs of PSCs. Among females considered to be adults (pulp width <35%), one female with a pulp width of 34% contained PSCs, while the remaining females with PSCs all had a pulp width below 28%. Among wild-born females, six had pulp widths between 21–26% (1+ year), and one female had a pulp width of <20% (2+ year). Six captive-born females had pulp widths of between 34% and 20% (1+ year), and two females had a pulp width <20 (2+ years). Two captive-born reproducing females had four age lines. This supports that females with a pulp cavity >35% are not reproductively mature.

The turnover was estimated to 66%, i.e., the percent mortality that corresponded to a stable population of wild-born mink [27]. The mean number of juveniles per wild-born adults, i.e., the fecundity divided by two, was 1.9. Accordingly, juveniles made up 0.66 (1.9/2.9: juvenile generation divided by juvenile generation plus adult generation) of the population at the beginning of each generation, defined as a turnover of 66%.

### 3.3. Mortality and Life Tables

The ages of 214 mink were determined. The overall juvenile/adult ratio (J/A) in the culled mink population was 1.7 (136/78). Only mink more than 4 months old (from September) could be separated into wild-born and captive-born.

Both wild-born (q_x_ = 0.70) and captive-born (q_x_ = 0.77) mink had high mortality within the first year, while the mortality rate in one-year-old and two-year-old individuals was relatively low; q_x_ in wild-born mink ranged between 0.38 and 0.56, and in captive-born q_x_ ranged between −0.30 to 0.41 (Table 2). Only five (3%) of the aged mink were determined as being more than four years old. The oldest individuals had five age lines and were considered to be 4.5 years old. No individuals were found with more than five age lines.

The average proportion of individuals dying per year in wild-born mink was (0.66 × 0.70) + (0.23 × 0.56) + (0.10 × 0.38) + (0.06 × 0.8) + (1 × 0.01) = 0.46 + 0.13 + 0.04 + 0.05 + 0.01 = 0.69. The average mortality per generation in captive-born mink was (0.63 × 0.77) + (0.19 × 0.41) + (0.11 × 0.30) + (0.14 × 0.69) + (0.04 × 1) = 0.49 + 0.08 − 0.03 + 0.10 + 0.04= 0.68.

## 4. Discussion

In a previous investigation of mink caught in the wild in Denmark, a comparison of the body length of captive-born mink and wild-born mink revealed a substantial number of mink escapes from Danish mink farms [7]. Hence, newly escaped farmed mink may bias studies of wild-born mink reproduction and demography and, thereby, the question of a self-sustainable population. This bias may lead to false conclusions regarding the demography, reproduction, and turnover and, henceforward, management actions for mink. In this study, wild-born and captive-born mink culled by hunters were separated in two groups before the examination of reproduction and mortality. There may be the bias that the hungriest mink will enter traps more than the well-fed ones. This may mean that captive-born mink, not able to catch prey, or juveniles may be more willing to enter the traps compared with wild-born adults. However, this is considered to be a minor bias as there appeared to be very little competition between mink, as the mink caught in the traps were generally well fed and in good health.

Pulp width in the canine teeth is used as a guideline for age, and it is species-specific [20,22]. However, in the age determination of mink in previous studies, authors referred to the pulp width of pine martens [15,21]. The annual layers (incremental lines), which appear in the canine tooth cementum, are useful as an age determining guideline and are widely used in the age determination of carnivores [20,22]. In mink, a dark incremental line is formed in the late summer and autumn, and a second line develops in individuals of approximately 1.5 years of age [22,23]. However, the development of the incremental lines can be uneven among terrestrial mammals in different areas, and adult individuals may lack age lines [22,23].

In the present study, pulp cavity filling showed a polynomial exponential growth, with stepwise filling of the pulp cavity during the first 10 months followed by a flattening of the modeled pulp filling curve (Figure 4B). Compared to pine martens, the pulp cavity of mink appeared to have a slightly slower centripetal growth, divided between juveniles and older pine martens at 10 months of age with a pulp cavity width between 16% and 32% (mean 24%). Danish mink at 10 months of age showed a pulp cavity width ranging from 27% to 34% (mean 30%). Hence, using the pulp width of pine martens on mink would assign a higher number of individuals as juveniles compared to adults, compared with if the accurate regression line for mink is used. In using both the actual mink pulp width versus age and incremental lines, the demography is considered to be more accurate. However, a detailed study of the incremental lines in aging mink is required in the future.

Normally PSCs degenerate (fade) after 7 months [13]; thus, staining of PSCs from previous years is not expected. Hence, all scars, both dark and faded, were interpreted as marks from fetal attachments of the current reproduction cycle. The proportion of barren females was relatively high (50% in wild-born and 42% in captive-born). The reproduction of Danish wild-born mink (mean litter size 7.6 and fecundity 3.8) was high compared to the litter size of wild-born mink ranging from four to seven kits as observed in other European studies [13,15,19,28].

In a study from Scotland, evidence of a density-dependent response in female fecundity was found, with a negative relationship between the fecundity and relative density of females. Mink culling leads to a younger, more fecund mink population, which is considered to be a compensation for the culling [15]. The high number of kits per female observed in this study may, therefore, be due to culling effects. High culling is also expected to reduce the number of barren females in a population [29]. However, this does not correspond with the relatively low percent of reproducing females found in the Danish wild-born mink population (50%) compared to the studies in Scotland (81%) by [15].

The mortality rate of both wild-born and captive-born mink (q_x_ > 0.70) was higher within the first year than in one-year-old and two-year-old individuals. In two-year-old captive-born mink, a negative mortality rate was found (q_x_ = −0.30) (Table 2). The negative mortality rate may reflect an influx of escaping farmed mink, indicating that the demography of farmed mink may not reflect the survival and mortality in the farmed population in nature but, more likely, merely the escape rates of young adults and adults from farms. Only five (3%) of the aged mink were more than four years old. The oldest individuals had five age lines and were considered to be 4.5 years (Table 2). The low number of adults may reflect the high hunting pressure or that older individuals are less likely to enter traps than younger animals [30]. The average number of individuals dying per year in the wild-born mink population was 0.69.

The J/A ratio was found to reflect the hunting pressure on three populations of mink: in the UK, South Harris and North Uist, and on an Estonian archipelago in the Baltic Sea [25]. High hunting pressure led to a relatively high J/A ratio, while low hunting pressure led to a low J/A ratio. In a study of two mink populations in North America, the harvest pressure affected the J/A ratio [24]. In Idaho, with a moderate hunting pressure, the J/A ratio was 1.47, compared to Alaska, with almost no hunting pressure, where the J/A ratio was 0.8 [24]. The overall J/A in the Danish feral mink populations (1.7) indicated a moderate to high hunting pressure.

Based on the fecundity of wild-born females, the turnover of wild-born mink was estimated to 66%. Compared to the yearly mortality estimated at 69%, these data point to a declining population of wild-born mink in Denmark, which is in agreement with national game bag records of mink. However, the yearly mortality is only marginally higher than the estimated turnover, and the balance between recruitment of the new generation and yearly mortality may tip with small changes in either the reproduction or mortality.

## 5. Conclusions

When mink on farms are moved out of their cages, e.g., during mating and pelting, they sometimes escape from the farms. Although the number of mink that escape from each farm may be low, this still results in around 30% of the mink caught by hunters in the wild being mink that were born on farms [7]. The concern was that this constant influx of escaped farm mink to the wild population contributes to maintaining the feral mink population in Denmark, rather than the reproduction of mink adapted to live in the wild.

The data showed that the wild-born mink population in Denmark does reproduce (mean ± SE litter size of 7.6 ± 0.9 and fecundity 3.8 ± 0.9). Hence, the population of feral mink in Denmark is self-sustaining without a continuous influx of captive-born mink escaped from farms. However, the escaping mink from farms may supply the wild-born mink population with additional individuals. The effect of farmed mink on the wild-born population depends on the survival rate and the ability of farmed mink to adapt to a life in nature, which is unknown at this time.

## Figures and Tables

**Table 1:** Summary of reproduction of 41 adult mink females; of these, 34 known to have died within 7 months post-partum were used to estimate the fecundity and percent reproducing females. Mean litter size ± SE (excluding barren), fecundity ± SE (including barren).

Reproduction	Wild-Born Females	Captive-Born Females
Litter size	7.6 ± 0.9 (range 5–11, *n* = 7).	5.9 ± 0.9 (range 1–10, *n* = 11)
Fecundity	3.8 ± 1.2, *n* = 14	3.4 ± 0.9, *n* = 20
Reproducing females	50%	58%

**Table 2:** Life table of wild-born and captive-born mink. Danish mink: *n* = number (percent) of mink in each age class; q_x_ = mortality rate. The start generation (0) was calculated from the fecundity of females (Table 2).

Generation	Wild-Born *n* = 80	Captive-Born *n* = 91	Wild-Born q_x_	Captive-Born q_x_
0 generation	61 (66%)	75 (63%)	0.70	0.77
4 month to 1 year	48 (60%)	47 (52%)	0.62	0.64
<1 year	18 (23%)	17 (19%)	0.56	0.41
<2 years	8 (10%)	10 (11%)	0.38	−0.30
<3 years	5 (6%)	13 (14%)	0.80	0.69
<4 years	1 (1%)	4 (4%)	0.100	1.00

## Data Availability

The data presented in this study are available upon request from the corresponding author.
